# Supplementary material for: Characterizations of a Class-I BASIC PENTACYSTEINE Gene Reveal Conserved Roles in the Transcriptional Repression of Genes Involved in Seed Development
Source: Curr Issues Mol Biol. 2022 Sep 7;44(9):4059–69. doi: 10.3390/cimb44090278 (PMC9497819; doi:10.3390/cimb44090278)

Supplementary Tables

Supple. Table. S1 The primer sequences used for qRT-PCR amplification.

Table S1 The primer sequences used for qRT-PCR amplification

Primer usage	Forward primer sequence (5'-3')	Reverse primer sequence (5'-3')
CjBPC1 RT-PCR	CCCATACCGGTTTGCTCTTG	ACCACATCCCCATCGGTAAC
CjBPC2 RT-PCR	TCCCACCGCCCTCAAAA	TCAGCCCCGGTGGACAT
CjBPC3 RT-PCR	GAAGAACGCAGCTGTTGAAGAA	TCCTCTATGGCTGCATTTTTTC
CjBPC4 RT-PCR	TTACTGATGCACCCCCAATGT	TTTAGTCCGCCTAGGCTTTGC
CjBPC5 RT-PCR	TGCAGCCGAGGGTCATG	TGGCCCAGTGATCCTTGAG
CjBPC6 RT-PCR	ACCCGGTATTGCTGCTGTTC	TCTCTGCCGGCTCAGACATA
GAPDH internal reference	GGGAATCCTTGGTTACACTGAG	ACCCCATTCGTTGTCATACC
CjBPC1 full-length cloning	AGCACAGTACTGCAATGCCT	AACAGACCAGATGCGATGGG
CjBPC2 full-length cloning	GCTTCAGTTTCGGCAGTCCA	AAACAGACCACGAGCACTGG
CjBPC3 full-length cloning	ATGGATGACGGTGGGAAAC	CTTGATGGTAATGTAGCGATTTG
CjBPC4 full-length cloning	ATGGATGCCAGTGGGCATC	CACGATATCCCAGTAGTACGCA
CjBPC5 full-length cloning	AGATGGATTTTTAGTGGTTTCCCT	CACGATATCCCAGTAGTACGCA
CjBPC6 full-length cloning	ATGGGGACACACCACGATAG	TTACTTAATTGTAACAAAC
CjBPC1 EXCLONE adaptor	tcagcagtcgaagagcATGGCTCAATCTGTAGTTTC	ttagcgtgtgaagagcCCTGATTGTCACAAACTTG
Transgenic validation PCR Hygromycin	TGACCTATTGCATCTCCCGC	ATTTGTGTACGCCCGACAGT
CjBPC1-F1/R1	CCCATACCGGTTTGCTCTTG	ACCACATCCCCATCGGTAAC
CjBPC1-F2/R2	CGAGATTGTGCGGTTTCAGA	GGTTTACCCAACTATCCCTCACAT
ACTIN reference in Arabidopsis	AAGGAGAAGAAGGAGAAGGAACAG	CAAGGAGTGCAATGACTGTGAGATA

AtSTK RT-PCR	GTTCTGATAGCACCAACACTAGC	ACTCATGCTTCTTGGACCTGATC
AtABI3 RT-PCR	AAGCAAAGCGACGTGGGTAA	GTGTGTCTCAGCTTCTTTTTTGG
AtFUS3 RT-PCR	GCTCACTTGCCGGCACTT	AACCGTCCAAATCTTCCATTCTT
AtLEC2 RT-PCR	TGGCAAGAGAGAGGTGGTTTTC	TCCTGTTGATCCTTGCCATCT
AtSTK ChIP-PCR	CGTCTGCGAAAAACCGAGCT	GGACCAATACCTTCATTGTACTTTGAA
AtABI3 ChIP-PCR	GCCTCCTTACTCACATACAAACCC	TCATCAGCGTCTCCACCGAGTATT
AtFUS3 ChIP-PCR	CGACGTATGATACTCCCGAAGGTGAT	ATCCCTTCCTTGCATTCAAGTGCC
AtLEC2 ChIP-PCR	CGCTCGCACTTCACAACAGTCC	TCATCACCGCCGCCATCTGC

Supple. Table. S2 Characteristics of the *BASIC PENTACYSTEINE* family in *Camellia japonica*.

TableS2 Characteristics of the BASIC PENTACYSTEINE family in *Camellia japonica*

Gene name	GeneBank ID	Length (aa)	Molecular weight (KD)	PI	Subcellular location
CjBPC1	OL631154	289	32.0	9.40	Nuclear
CjBPC2	OL631155	298	32.6	9.67	Nuclear
CjBPC3	OL888113	312	34.9	9.03	Nuclear
CjBPC4	OL888114	332	37.1	9.53	Nuclear
CjBPC5	OL888115	305	34.1	8.81	Nuclear
CjBPC6	OL888116	232	25.1	9.68	Nuclear

Supplementary Figures

Figure. S1: the details of conserved motifs in BPC proteins. The upper panel indicates the distribution of motifs that are presented by colored boxes. The lower panel lists the detailed conserved amino acids of each motif.

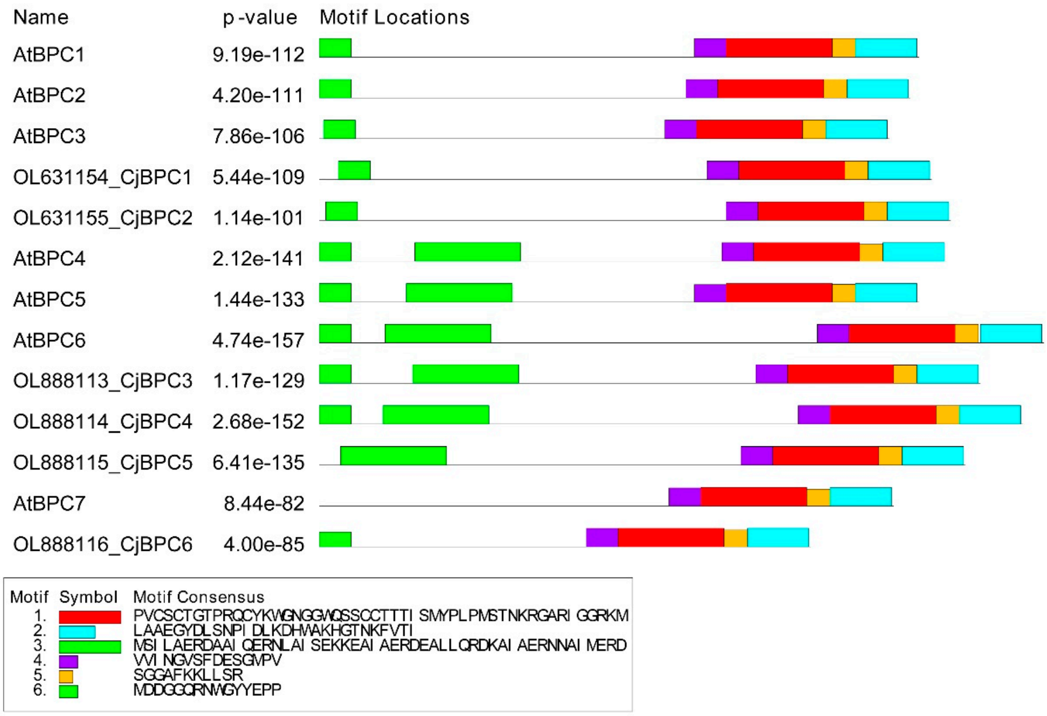

Figure. S2 Expression profiles of CjBPC genes in different tissues of *Camellia japonica.* The expression *CjBPCs* in differe floral tissues of *Camellia japonica*. The expression is measured by the fragments per kilobase per million (FPKM). Three biological replicates are used for the expression analysis as described. CjYL, young leaves; CjFB, floral buds; CjPE, petal; CjEM, fruit endocarp; CjSD, Seed.

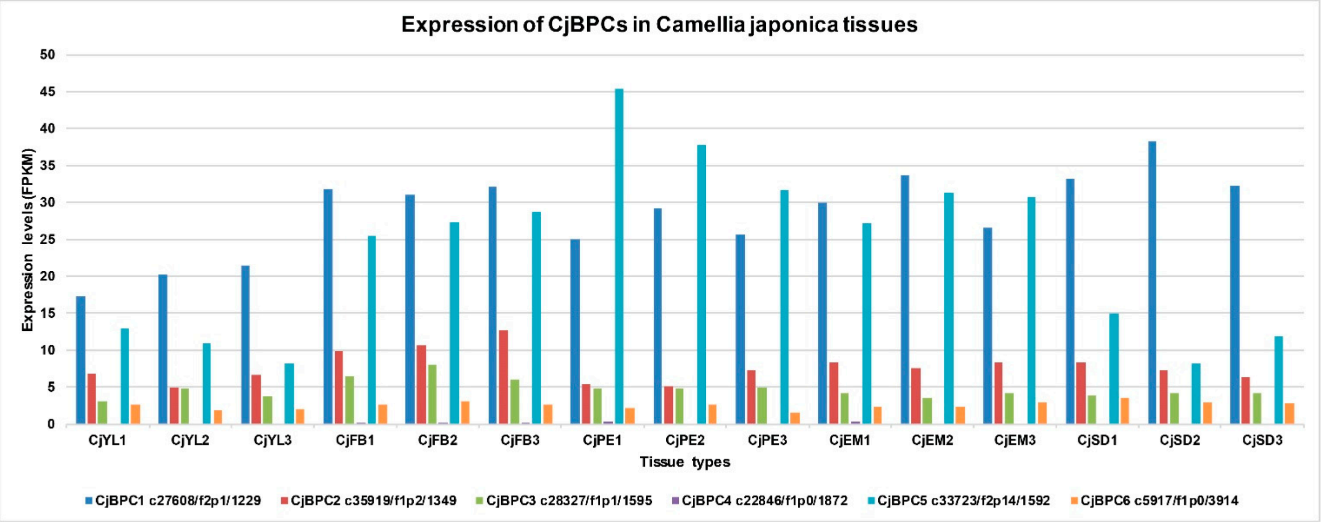

Figure. S3 Overexpression of *CjBPC1* in Arabidopsis and molecular validation of transgenic lines. A, the diagram of overexpression construct for Arabidopsis transformation. **B,** the PCR verification positive T1 transgenic lines. **C,** the relative expression of CjBPC1 in different transgenic lines using gene-specific primers.

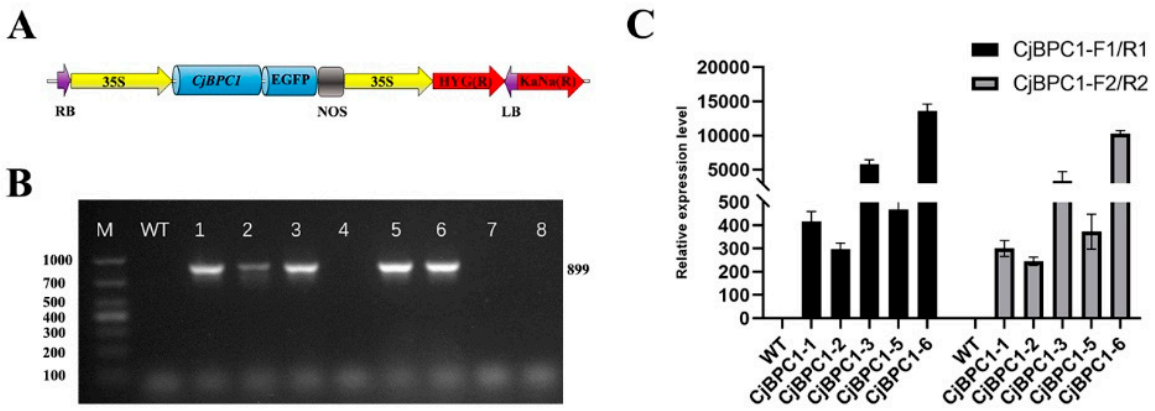

Supplement: Supplementary file 1 [file cimb-44-00278-s001.zip › cimb-1891944-supplementary.pdf]
